# 

**Published:** 1895-07-27

**Authors:** 


**Notices of Births, Deaths, and Marriages** are inserted in this column at a charge of 2s. 6d. for an announcement not exceeding four lines.

**Notice to Advertisers and Subscribers.**

All Advertisements, Orders for Copies of The Hospital and Business Communications should be addressed to **The Manager, NOT TO THE EDITOR**, The Hospital, 428, Strand, London, W.C.

The Cost of Subscription, post free, is as follows:—Per week, 2½d.; per quarter, 2s. 8d.; per half-year, 5s. 3d.; per annum, 10s. 6d. Foreign:—Per Annum, 15s. 2d.; payable, in all cases, in advance.

### NOTICE TO CORRESPONDENTS.

All MS., Letters, Books for Review, and other matters intended for the Editor should be addressed The Editor, The Lodge, Porchester Square, London, W.

The Editor cannot undertake to return rejected MS., even when accompanied by stamped directed envelope.

**"Burdett's Hospital Annual," 1895,**
Now Ready.

Sixth year of publication. 950 pp. Scarlet cloth, gilt lettered. Price 5s. A most complete guide to British, Colonial, Indian, and American Hospitals, Dispensaries, Nursing and Convalescent Institutions, and Asylums. A few copies of the "Annual" for 1894 may still be obtained on application to the Publishers, The Scientific Press (Limited), 428, Strand, London, W.C.

**NOTICE.—Vol. XVII. of "The Hospital" (October, 1894, to April, 1895), in handsome cloth binding, is on sale at the Office of this Journal. Price 6s. Cases for binding the Numbers contained in this Volume, 1s. 6d. Index to Vol. XVII., 2½d., post free.**

**The Monthly Part for August will be ready on August 1st, price 6d.; by post, 8d.**

### Scraps and Gleanings.

Donations to the amount of £3,825 were announced at the festival dinner of the Poplar Hospital for Accidents.

Over 5,000 ladies volunteered their services in London on the occasion of the twenty-second observal of Hospital Saturday.

A bust to Ludwig Trante, the eminent clinician, of Berlin, has been placed in the garden of the Charité Hospital, Berlin.

Mr. Llewelyn Howell has presented a valuable oil painting of a lifeboat during rescue work, to the Swansea Hospital.

Medical missions in India are assuming so extensive a character that a quarterly journal has now been started among them.

The Deighton, Sheepridge, and surrounding districts' fourteenth annual demonstration in aid of the Huddersfield Infirmary took place last week.

The West End Hospital for Diseases of the Nervous System, Paralysis, and Epilepsy has received a donation of £100 from the Worshipful Company of Grocers.

The Japanese Government have voted a sum of money to their eminent countryman, Dr. Kitasato, to further his researches. He is at present engaged in carrying out a series of experiments in leprosy.

The third annual Hospital Sunday, organised by the Uckfield Volunteer Fire Brigade and Friendly Societies, was held recently in favour of the funds of the Sussex County Hospital, Brighton Eye Infirmary, and Uckfield Cottage Hospital.

One hundred pounds has been bequeathed to the Norfolk and Norwich Hospital under the will of the late Mr. Robert Fitch, and £100 under the will of the late Rev. Williams Fellowes, towards the Leicester Perpetual Endowment Fund.

The Académie de Médécine has been presented with a report on the chemical richness of oysters in bromide, iodine, fluorine, and in phosphorus. After the late unpleasant suggestions regarding these delicacies, it is pleasant to hear their character vindicated scientifically.

A strong temperance movement has begun in France, based on the observations of physicians to lunatic asylums more especially. The consumption of alcohol has doubled itself in France in less than half a century, and absinthe especially is held responsible for an increase of epilepsy.

An offer has been made to the London County Council to take on lease the remainder of the surplus land in the Coldharbour Lane, with a view to erecting thereon a general hospital for South London. It is conjectured that this site is sufficient for such a hospital capable of containing 200 beds.

The special appeal made by the Dewsbury and District Infirmary Board, and commenced by the president, has not been supported as we would venture to observe it ought to have been. This appeal, which was circulated locally in the papers, brought in only the modest response of £1 15s.

The annual meeting of the subscribers to the Adelaide Hospital, Dublin, has now been held. During the period under review 1,200 intern and 1,500 extern patients have been treated during the year. No less than £1,273 has been received through the earnings of trained nurses connected with the institution.

During the late sultry days in July the hospitals of the Metropolitan Asylums Board have become unduly crowded. The Board had to write to the poor law authorities in London to urge them to send only the severe cases and those patients who, in consequence of their surroundings and conditions of life, were most in need of hospital treatment.

An adjourned conference of the local authorities comprised within the Preston, Fylde, and Garstang Union has now taken place, when it was resolved that a committee be formed to consider all details, consisting of one representative for each district for every £50,000 of rateable value. A petition is now to be presented to the County Council with regard to this question.

The annual report of the Swansea Hospital shows how the cost of the institution has increased from £4,136 to £4,648, in spite of the economy exercised, and how this had been met by a fortunate increase in the receipts, principally from a remarkable advance in the workmen's contributions, from Miss Talbot's donation of £800, Madame Patti's concert receipts of £352, and Mr. Henry Studt's fête, which produced £907.

The Mauldeth Hospital for Incurables (Manchester) has held its annual sale of needlework in the presence of a large company.

The session of the Pathological Society closed in London with the annual meeting. A prosperous condition is shown in the report of this society.

A special appeal for extended financial aid for the Hertford General Infirmary has been started in the local newspapers and is meeting with a hearty response.

The Worshipful Company of Grocers has contributed £100 to the funds of the Metropolitan Convalescent Institution, whose office is at 32, Sackville Street, W.

The fourteenth annual report of the Uckfield Cottage Hospital states that 34 cases had been admitted into the institution during the year. The subscription list has again decreased.

The annual church parade of the friendly societies of Kingsthorpe in aid of the Northampton General Infirmary has just taken place with a successful issue, and the institution has benefited financially therethrough.

The deficit existing on the accounts of the Wellington and District Cottage Hospital has been cleared off and a small balance left in favour of its funds, through the auspices of a very successful bazaar inaugurated and held recently.

The report of the Managing Committee of the Birmingham Hospital for Women states that the number of new out-patients shows an increase of 179 on the previous year. Lady Dudley has presented the hospital with a donation of £25.

The members of the Derbyshire Children's Thursday Committee had their annual outing this month, the places selected for visitation this year being Chatsworth, Haddon, and Bakewell. The collections of this society amounted last year to £206.

A committee, appointed by the Glamorgan County Council, held an inquiry at the Pontypridd Police Court last week into the question of erecting an isolation hospital for the use of the inhabitants of the urban districts of Pontypridd and Caerpheldy.

Next summer there is to be an International Congress at Bordeaux for the "Protection of Infancy," whereat, we are informed, "the moral, legal, and physical sides of the subject" will be submitted for discussion; and, further, "The decline of parental authority and its delegation into other hands" will be enlarged upon.

The committee of the Children's Free Holiday Home at Southend remind us in their circulars of the opportunity afforded the charitable public through this institution of giving a free day at the seaside to the small waifs and strays of London. A donation of £1 gives ten little ones a like treat, including a dinner, tea, and railway fare.

The Mazawattee Tea Company issue a little handbook to Great Britain and Ireland that will prove very useful to intending holiday-makers. It gives concise information of most places of interest within the Kingdom, and is prettily illustrated. As it can be obtained from all the agents of the Company, travellers can obtain it without difficulty, and it is well worth possessing.

A German paper gives us an account of a new danger menacing the welfare of doctors which has lately shown itself in Munich. This is presented in the appearance of "medical corporations" lately inaugurated, and advertised as giving their treatment at extremely low rates. This will receive the serious consideration of the local public, it is feared, and strike a serious blow to the medical practitioners.

The Hull Royal Infirmary points out that 60 cases have been received in the wards during the year from Grimsby and its immediate neighbourhood, with a residence of 1,494 days, and at a cost of £186, whilst during the same period there was received in contributions the sum of eight guineas. These figures speak for themselves, and the secretary of the hospital has issued a general appeal for subscriptions from the public of Grimsby.

One hundred and fifty-one in-patients were admitted to the Devonshire Hospital and Buxton Bath Charity during the last half-year, under the powers of the governors of the Cotton Districts Convalescent Fund. This so far signifies the extension of the benefits of the hospital to probably needy sufferers, chiefly from diseases of a rheumatic character, among the operatives of the cotton manufactures who appear to be especially sufferers from their employment.

### The Student of Medicine.

### Appointments.

E. Allan, L.R.C.P. and S.Edin., late House Surgeon Glasgow Royal Infirmary, appointed Assistant Medical Officer Barony Parish Hospital, Barnhill, Glasgow. F. J. Allan, M.D.Edin., appointed Examiner in Medical Jurisprudence and Public Health at the University of Aberdeen. J. W. Ballantyne, M.D.Edin., appointed Examiner in Midwifery at the University of Aberdeen. Dr. Bell, appointed Medical Officer of Health to the Lowestoft Town Council. Dr. Berry, appointed Medical Officer for the Enniskillen Dispensary Distribt. J. R. Bradford, M.D., D.Sc.Lond., F.R.S., appointed Examiner in Medicine at the University of Aberdeen. W. D. Buncombe, L.R.C.P.Lond., M.R.C.S.Eng., appointed Medical Superintendent of City of London Infirmary, Bow. W. E. Bond, M.R.C.S., L.R.C.P., appointed Resident Medical Officer to the Brighton and Hove Hospital for Women. E. Bryne, L. and L.M.K.Q.C.P.I., L.R.C.S.I.,, appointed Medical Officer and Medical Officer of Health Bannow Dispensary District, co. Wexford. R. J. H. Gibson, M.A.Aberd., appointed Examiner in Botany at the University of Aberdeen. G. G. Gidley, M.R.C.S.Eng., L.R.C.P.Lond., L.S.A., appointed Medical Officer and Public Vaccinator for the Cullompton and Butterleigh and Kentisheave and Blackboro' Districts of the Tiverton Union T. W. Griffith, M.D.Aberd., appointed Examiner in Anatomy at the University of Aberdeen. J. Hunter, F.I.C., F.C.S.Edin., appointed Examiner in Chemistry at the University of Aberdeen. A.

### THE STUDENT OF MEDICINE—*continued.*

Keith, M.D., F.R.C.S., appointed to the Demonstratorship of Anatomy in London Hospital Medical School. E. W. D. Kite, M.B., M.R.C.S., L.S.A., appointed Surgeon under the Factories Acts to the Oulerton District, Sheffield. J. T. Maclachlan, M.B., C.M.Glasg., appointed Assistant Medical Officer to Lanark District Asylum, Hartwood. L. J. Minter, M.D.Brux., M.R.C.S., L.R.C.P., L.S.A., appointed Medical Officer of the Workhouse of the Uxbridge Union, and Certifying Factory Surgeon for the Uxbridge District. F.W. Mott, M.D.Lond., F.R.C.P., M.R.C.S., appointed Pathologist to the London County Asylum. Dr. Phillips, appointed Medical Officer of Health to the Market Harborough Rural District Council. Dr. W. Randall, appointed Medical Officer of Health to the Maesteg Rural District Council. J. Lorrain Smith, M.A., M.D.Edin., appointed Examiner in Pathology at the University of Aberdeen. J. A. Erskine Stuart, L.R.C.P.Edin., L.R.C.S.Edin., appointed Medical Officer of Health for the Borough of Batley. W. H. Thompson, F.R.C.S., appointed Examiner in Physiology at the University of Aberdeen. J. A. Thomson, M.A.Edin., appointed Examiner in Zoology at the University of Aberdeen. F. Warner, M.D.Lond., appointed Examiner in Materia Medica at the University of Aberdeen. J. Ryland Whitaker, B.A., M.B.Lond., F.R.C.P. Edin., L.R.C.S.Edin., appointed Lecturer on Anatomy at Surgeons' Hall School of Medicine, Edinburgh. John C. Ogilvie Will, M.D.Aberd., appointed Examiner in Surgery at the University of Aberdeen. Dr. Wills, appointed Medical Officer of Health to the Blythe and Cuckney District Council. T. Wilmot, L.R.C.P.Lond., M.R.C.S., appointed Honorary Medical Officer to the Bradford Infirmary. J. Sutherland Mackay, M.A., M.D., D.P.H., appointed Medical Officer of Health for the Burgh of Kirkcaldy.

### VACANCIES.

The following vacancies are announced this week, the amount of salary in each case being given after the name of the institution:

#### Resident Medical Officers.

Chichester Infirmary.—House Surgeon. Salary £80 per annum, with board, lodging, and washing. Applications to the Secretary by August 12th.

Great Northern Central Hospital, Holloway Road, N.—Junior House Surgeon. Appointment for six months. Board, lodging, and laundry provided. Applications, on forms provided, to be sent to Lewis H. Glenton Kerr, Secretary, by July 29th.

Morpeth Dispensary.—House Surgeon, doubly qualified, unmarried. Salary, £120 per annum. Applications to N. J. Wright, Morpeth, Northumberland, by July 31st.

Paddington Green Children's Hospital, London, W.—Assistant House Surgeon. No salary. Board and residence provided. Applications to the Secretary by July 26th.

Chester General Infirmary.—Visiting Surgeon, doubly qualified. Appointment for two years. Salary to commence £90 per annum, with residence and maintenance in the house. Applications to the Chairman of the Board of Management, Secretary's Office, 29, Eastgate Row North, Chester, by July 30th.

#### Non-Resident Medical Officer.

Hospital for Women, Soho Square.—Non-Resident Assistant House Physician. Appointment for three months. Applications to the Secretary by July 31st.

### CHARING CROSS HOSPITAL MEDICAL SCHOOL.—ANNUAL DISTRIBUTION OF PRIZES.

The distribution of prizes to the successful students of Charing Cross Hospital took place on Friday, July 18th. Sir Edward Lawson took the chair, and presented the prizes to the successful students. The prize winners included the following students: Mr. E. P. Jones, Mr. D. C. Rees, Mr. W. Green, Mr. C. B. Wagstaffe, Mr. W. H. Unwin, Mr. J. E. Humphreys, Mr. W. E. Morgan, Mr. W. C. Bosanquet, Mr. C. Hudson, Mr. E. L. Lilley. The Dean, Dr. Stanley Boyd, read his report on the condition of the school, and referred to the losses sustained by the hospital through the death of Dr. Hack Luke and the respective resignations of Dr. Mott and Mr. Chalmers Mitchell. He attributed the falling off in the number of students in their own as well as in the other London hospitals to the unsettled prospect of the teaching University of London. Sir Edward Lawson, in addressing the company and the students, congratulated them on the efficiency of the work performed in the wards, and contradicted the report that had been going the round of the papers, saying that Charing Cross Hospital was about to move to the other side of the river. So far from this being the case, he urged on the authorities the acquisition of Toole's Theatre, an increase in the present ward accommodation, and the institution of new resident appointments. More money was wanted, and it ought to be got. The work of the hospital could best be performed in the very heart of the great city, where it now stood. Dr. Mitchell Bruce and Mr. Bloxam proposed and seconded a vote of thanks, and the ceremony terminated.

### UNIVERSITIES AND COLLEGES.

#### Royal College of Surgeons of England.

The following gentlemen were at the quarterly meeting of the Council on Thursday, July 11th, elected to the various offices for the ensuing year:—

Hunterian Professors.—Charles Stewart, M.R.C.S.Eng., Conservator of the College Museum (St. Thomas's Hospital), subject of lecture not stated; John Alfred Coutts, M.B.Cantab., M.R.C.S.Eng. (St. George's Hospital), on Infantile Syphilis; Leonard Erskine Hill, M.B.Lond., M.R.C.S.Eng. (University College Hospital), on Cerebral Pressure and the Cerebral Circulation.

Arris and Gale Lecturer.—Ernest Henry Starling, M.D. and B.S.Lond., M.R.C.S.Eng. (Guy's Hospital), on the Physiological Causation of Dropsy.

Erasmus Wilson Lecturer.—Walter George Spencer, F.R.C.S.Eng., M.B. and B.S.Lond. (Westminster Hospital), on the General Pathology of Bone.

Pathological Curator.—James Henry Targett, M.B., B.S., and M.S.Lond., F.R.C.S.Eng. (Guy's Hospital), re elected.

Anatomical Assistant in the Museum.—Richard Higgins Burn, B.A.Oxon, re-elected.

#### Royal College of Surgeons in Ireland.

School Prizes.—The Barker anatomical prizes have been awarded to N. H. Alcock and C. J. Patten (equal). A special prize has been awarded to W. J. Sweeny. The Carmichael Scholarship has been won by F. J. Palmer. In Surgery the gold medal has been won by L. McDowell; the silver by G. A. Robinson. In Practical Histology the first prize was gained by D. Hadden; the second by H. Hall. In Practical Chemistry P. A. Frazer gained the first prize, and C. C. Meck and W. J. Anglim were equal for the second prize. In Public Health and Forensic Medicine A. I. Eades gained the first prize and Miss C. L. Williams the second. In Materia Medica R H. D. Pope won the first prize and G. R. McDonald the second. In Practical Pharmacy S. R. Godkin obtained the first and M. Gavin the second prize. In Biology M. Gavin gained the first prize and W. McLorinan and W. J. Anglim were equal for the second.

#### Royal Colleges of Physicians and Surgeons, Ireland.

The preliminary examination for the commencement of medical studies of the Royal Colleges of Physicians and Surgeons in Ireland, for the session 1895-96, will be held on Tuesday and Wednesday, October 1st and 2nd. Prospectuses can be had on application to the Registrar, at the Royal College of Surgeons, St. Stephen's Green W., Dublin.

